# Supporting managerial decisions: a comparison of new robotic platforms through time-driven activity-based costing within a value-based healthcare framework

**DOI:** 10.1186/s12913-025-12598-9

**Published:** 2025-03-29

**Authors:** Stefano Landi, Gianluca Maistri, Luca Piubello Orsini, Chiara Leardini, Sarah Malandra, Alessandro Antonelli

**Affiliations:** 1 Department of Management, University of Verona, Via Cantarane 24, Verona, 37129 Italy; 2 Department of Surgery, Dentistry, Pediatrics and Gynecology, Urology Unit, University of Verona- Azienda Ospedaliera Universitaria Integrata Verona (AOUI), Piazzale Aristide Stefani, 1, Verona, 37126 Italy

**Keywords:** Technology assessment, Value-based healthcare, Time-driven activity-based costing, Managerial decision-making, Robotic platforms, Robotic-assisted radical prostatectomy

## Abstract

**Background:**

The advent of novel robotic platforms requires that managers base their decisions on the value these platforms generate. This study showcases how micro-costing methodologies can assist managers in the decision-making process regarding the implementation of new robotic platforms within the value-based healthcare (VBHC) framework.

**Methods:**

We applied time-driven activity-based costing (TDABC) to evaluate cost disparities between the da Vinci and Hugo robotic systems for robot-assisted radical prostatectomy (RARP). Data were collected from consecutively enrolled patients with organ-confined prostate cancer. Basic cost information was gathered from Azienda Universitaria Integrata di Verona’s finance and pharmacy departments. We conducted cost and sensitivity analyses to evaluate the most cost-sensitive parameters.

**Results:**

The da Vinci system incurred higher total costs for RARP than the Hugo system (€4,97.21 vs. € 3,511.73, *p*-value < 0.001) However, excluding surgical kit costs, the da Vinci platform proved less expensive (€1,481.18 vs. €1,926.18, *p*-value < 0.001). Sensitivity analyses identified surgical kit costs as the most influential parameter, followed by surgical duration and platform costs.

**Conclusions:**

This study highlights the importance of micro-costing practices in supporting managerial decisions within a VBHC framework. When clinical outcomes are equivalent, the value of robotic platforms is related to cost savings. By using TDABC and sensitivity analyses, managers can pinpoint critical activities and parameters to optimize the effective adoption of new platforms.

**Supplementary Information:**

The online version contains supplementary material available at 10.1186/s12913-025-12598-9.

## Background

Robotic technology has revolutionized global patient care [1], introducing significant advancements in diagnosis, surgical procedures, and rehabilitation [2]. From its first documented healthcare application in the 1980s, robotic systems have evolved rapidly, enhancing precision [3], dexterity, and minimal invasiveness in surgeries [4]. These advancements reduce patient trauma and recovery time [5], particularly in urological procedures such as robot-assisted radical prostatectomy (RARP) [6].

Currently, three surgical techniques exist for radical prostatectomy: open surgery, laparoscopic surgery, and RARP. Of these, RARP has rapidly supplanted traditional open and laparoscopic techniques in many countries owing to its technical advantages and superior clinical outcomes [5]. For instance, the adoption of RARP in England’s National Health Service increased from 5% in 2006 to 88% in 2018, mirroring trends in the United States [7]. This widespread adoption is driven by the technical advantages of the technology as well as the safety and efficacy of robotic surgeries [8, 9]. Research conducted all across the world has demonstrated the advantages of RARP over other forms of radical prostatectomy, including Open Radical Prostatectomy (ORP) and Laparoscopic Radical Prostatectomy (LRP). Although more costly, RARP has been found to have better clinical results in radical prostatectomy, therefore justifying its use. For instance, Parackal et al. [10] found RARP to be cost-effective compared with ORP in their analysis of a Canadian population over a 10-year time horizon. In a similar fashion, in the UK, Labban et al. [11] found RARP to be cost-effective compared to both ORP and LRP. The results were mainly driven by the reduction in the rate of biochemical recurrence, although it’s crucial to emphasize that, contrary to Parackal et al. [10], this study showed RARP to be more costly when compared to ORP but less costly when compared to LRP. In the Netherlands, Lindenberg et al. [12] estimated the cost per operation, finding LRP to be less costly than RARP. However, based on a 7-year time horizon, it has been assessed that RARP is more cost-effective than LRP, largely because of the enhanced urinary functioning of RARP patients compared to LRP.

Despite these promising avenues, implementing this technology can be challenging for healthcare organizations. From a managerial perspective, these technologies are increasingly essential for addressing the complexities of modern healthcare systems, such as rising service demands, and demographic changes, while simultaneously requiring careful resource allocation due to the sector’s inherent budgetary constraints [3, 13]. The significant initial capital investment required for robotic systems [14] and ongoing maintenance costs [7] need the apex of healthcare organizations, characterized by their professional bureaucracy structure [15, 16], to make strategic decisions that suitably ensure the integration of professional knowledge and financial boundaries to converge the maximization of patient outcomes and organizational sustainability.

In this context, the recent democratization of robotics within this sector [17] further complicates the decision-making landscape for healthcare organizations’ managers. While the Da Vinci Surgical System (Intuitive Surgical Inc., Sunnyvale, CA, USA) has been established as the gold standard in (urological) robotic surgery for nearly two decades [18, 19], new platforms with different characteristics such as a modular systems, consisting of four separate arm carts and an open console with novel hand controls in a “pistol-like” style have been developed [20]. Such an example is that of Hugo RAS system (Medtronic, Minneapolis, MN, USA). which has witnessed an increasing global adoption in the last years [21, 22].

While this democratization process holds the potential to introduce competitive pressures in the market, thereby theoretically mitigating cost-related challenges, this greatly entangles the task of selecting the most suitable platform to align with the organization specific clinical and financial objectives. It is against this background that it now becomes crucial for managers to develop and employ techniques to accurately measure and evaluate the value [23] generated by implementing different robotics platforms.

Value, defined as “health outcomes achieved per dollar spent” [24], forms the basis of Value-Based Health Care (VBHC), an approach that seeks to engage both administration and medical staff in creating value for the patient by integrating cost and quality perspectives [25].

Implementing VBHC successfully requires precise assessment of real healthcare costs by employing methodologies that accurately measure resource consumption per patient [26]. This precision enables managers to comprehensively understand the value generated by a platform or clinical pathway and the respective inefficiencies [27].

Time-driven activity-based costing (TDABC) is widely regarded as the most suitable tool for implementing VBHC [28, 29], as it allows managers to measure actual costs by evaluating the time and resources required for each activity in a patient’s care pathway [30]. TDABC simplifies cost analysis by estimating only two parameters: the unit cost of resource inputs and the time required for each activity [31]. Studies have demonstrated TDABC’s ability to identify inefficiencies [32], improve process efficiency [30], and reduce costs while maintaining or enhancing patient outcomes [23, 32]. These applications in surgery primarily compare robotic surgery with traditional laparoscopic or open surgical procedures. However, researchers have given limited attention to how TDABC support managerial decision on technology adoption. In this regard, our study is novel in its focus on comparing robotic platforms in a context where the options of technologies are getting wider. As new robotic platforms enter the market, local hospital administrators, ministries of health and governing bodies could struggle to assess whether the costs of these technologies are reasonable, worthy of ongoing investment and able to inform future strategic decisions [9].

These decisions, following the VBHC, need to be based on scientific and empirical evidence for maximising the patient health and the efficient use of resources. As far as we know there are only a few studies comparing two robotic platforms [21]. In particular there are no studies applying a micro costing approach that take into consideration patient data level and engage clinicians in their analysis.

To address this research gap, we conducted a comparative cost analysis in a urologic surgery setting using two robotic platforms through the TDABC. Although TDABC studies typically focus solely on cost determination at the patient level, this study extends the investigation through the development of sensitivity analyses from the main cost-related factors associated with each platform.

This study identifies the key parameters that influence the value of each robotic platform, given equal clinical outcomes. This information will assist hospital managers in their decision-making processes. To achieve this objective, we analyzed the case of the Azienda Universitaria Integrata di Verona (AOUIVR), a public general hospital in Italy, which serves as a compelling case. AOUIVR recently participated in a clinical trial comparing robot-assisted radical prostatectomy executed with two surgical platforms: Da Vinci RARP (DV-RARP) and Hugo RARP (H-RARP). This provided a unique dataset for our comparative analysis, which is detailed in the following sections.

## Methodology

### Patients and study design

This study is based on the Comparison of Outcomes of Multiple Platforms for Assisted Robotic surgery—Prostate (COMPAR-P) trial, which received approval from the local ethical committee (*Comitato Etico per la Sperimentazione Clinica delle Province di Verona e Rovigo*, approval code 4038CESC) of the Veneto Region. The trial was registered at ClinicalTrials.gov (USA National Library of Medicine) under code NCT05766163 on March 10, 2023. All participants signed a dedicated informed consent form during the recruitment process. The details of the study, including inclusion criteria, clinical conditions, and period, have been described elsewhere [33, 34].

The COMPAR-P is a monocentric, post-market clinical follow-up study promoted by the Urology Unit of Azienda Ospedaliera Universitaria Integrata Verona (AOUIVR). A total of 100 patients with organ-confined prostate cancer were consecutively enrolled, with 50 patients assigned to RARP performed using the da Vinci system (DV-RARP) and 50 assigned to RARP using the Hugo RAS system (H-RARP). Enrollment began in March 2023, and the sole exclusion criterion was a patient’s refusal to undergo surgery with a robotic platform. The two groups were balanced in terms of demographic and clinical characteristics, except for minor differences observed in the Charlson Comorbidity Index and Briganti score [33].

The choice of robotic platform for each patient was determined according to platform and instrument availability. Surgical procedures were conducted according to standard clinical practice and no variation in technique depending on the platform. The surgical team underwent specific training for H-RARP, whereas DV-RARP was already the standard procedure at AOUIVR. Theater staff received three days of intensive training at the ORSI Academy in Melle, Belgium. The procedures were performed by two console surgeons who had previously completed over 500 DV-RARP operations. Notably, neither surgeon had any prior experience with the Hugo RAS system before this study.

Intraoperative performance and timings differences between DV-RARP and H-RARP have been analyzed in previous research [33]. Although the current study focuses on comparing the costs of the two procedures, prior studies indicated that DV-RARP was completed in a shorter period than H-RARP. However, both platforms achieved comparable surgical outcomes [33, 35].

### Time-driven activity-based costing

Time-Driven Activity-Based Costing (TDABC) simplifies the traditional ABC method by focusing on just two parameters: the unit cost of resource inputs and the time required for each activity. In addition, it opens up the possibility of engaging the clinical world in costing methodology, bridging the gap between the clinical and administrative worlds.

In detail, the TDABC analysis followed the 8-step framework proposed by Etges and colleagues [23] and the standardized framework from the TDABC in Healthcare Consortium [36]. The first step involved selecting the technology to be evaluated, specifically comparing the Hugo and da Vinci platforms for radical prostatectomy, as introduced in the previous section.

**Fig. 1:**
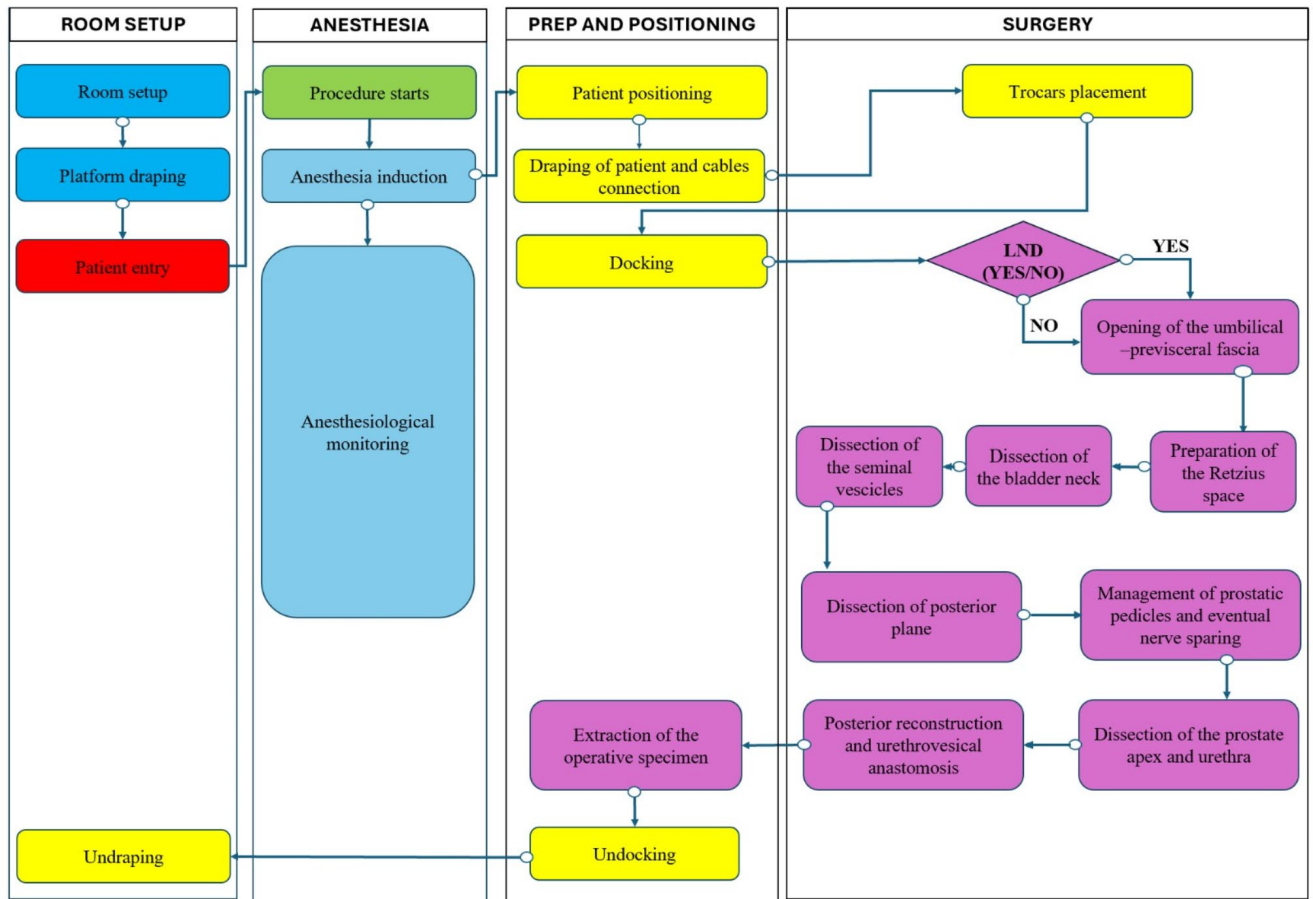
Process map for RARP. Legend: the color schemes represent the type of personnel involved. Nurses are always present throughout the procedure. Blue = only nurses; Light blue = senior anesthesiologist (+ junior anesthesiologist) + nurse anesthesiologist; yellow = 1 senior surgeon (+ 2 junior surgeons); purple = 2 senior surgeons (+ 2 junior surgeons)

The second step was to map the care delivery chain and create a process map of all the activities. Typically, the care delivery value chain (CDVC) outlines the clinical pathway, requiring a clearly defined start and end point for each patient. It identifies all activities involved in the care process and develops a detailed process map for each key activity. In this study, the CDVC focused on steps in which platform differences in time and cost were pronounced, namely the surgical procedure (case-time), the postoperative hospitalization (length of stay), and a follow up of 30-day to consider readmission events. The surgical procedure was detailed in a process map (see Fig. 1). Using contextual observations and three workshops with surgeons, nurses, and pharmacists, the clinical team [36] validated a map comprising 19 micro-activities (Fig. 1). Following previous literature [37], we grouped four macro-activities: “room setup,” “anesthesia,” “prep and positioning,” “surgery” and “console time”.

Figure 1 illustrates the composition of each micro-activity. Room setup comprises “room configuration,” “draping of platform,” and “undraping of platform.” The anesthesia encompasses the procedure and the latency period for its effect. Prep and positioning involves “patient positioning,” “draping of patient and cable connection,” “docking,” “extraction of the operative specimen,” and “undocking.” The surgical procedure is divided into: “trocar placement,” “lymph node dissection (LND),” “opening of the umbilical-prevesical fascia,” “preparation of the Retzius space,” “ dissection of the bladder neck,” “dissection of the seminal vesicles,” “dissection of the posterior plane,” “management of prostatic pedicles and eventual nerve sparing,” “dissection of the prostate apex and urethra,” and “posterior reconstruction and ureterovesical anastomosis.” Console time begins with “LND” and ends with “anastomosis.”

The third step involved identifying the resources used during the surgical procedure. Through meetings with surgeons, anesthesiologists, pharmacists (responsible for acquiring materials), and nurses, two main categories of resources were identified: human-related and theater-related. Human-related resources included the professionals involved in separate activities of surgery. Theater-related resources included the platforms, surgical kits (containing all necessary instruments), and operating room costs (see Table 1 in Appendix).

To proceed, the acquisition costs for each resource group were retrieved from data provided by the hospital’s financial and pharmacy departments. Costs for renting robots and acquiring materials were obtained through the hospital’s tender process, consistently with previous cost-related studies in healthcare [38, 39].

This has allowed us to proceed to the next stage of the process, which is to estimate the practical capacity of each resource and calculate the capacity cost rate.

The practical capacity is the effective capacity at which a certain resource can actually operate. We consider practical capacity, rather than a theoretical one, as the former takes into consideration time spent on downtime (for equipment and facilities) and breaks (for personnel) [23]. As far as the theoretical capacity goes, we considered 12 h a day for the platforms -after consultations with the surgery team- while for personnel we considered the hours given by the financial department. The Capacity Cost Rate (CCR) is calculated by dividing the cost of resources by the practical capacity of each personnel resource or structure department. As such, in line with most research [40] we evaluated the practical capacity of each personnel resource and platform- at 80% of their theoretical capacity. To see details regarding the CCR for each resource see Table 1 in the Appendix for CCR details).

The sixth step estimated the time each resource spent with patients at each process step using chrono-analysis [23]. This method was based on in situ observations when healthcare professionals measured resource time during surgery using a smart app. Data for each patient were prospectively recorded in a Research Electronic Data Capture (REDCap) database by a dedicated investigator who was not involved in the procedure. Time equations were then estimated for each activity. We analyzed which clinical parameters (e.g., age, BMI, PSA levels) could influence activity duration and, consequently, impact the cost of each individual activity. The only factors that significantly affect the time equation were (i) the need for the patient to undergo LND for the surgery activity (40 min longer) and (ii) the different platform used (which resulted in a total increase of 40 min across all activities). The LND was conducted on a subset of patients (23 for DV-RARP and 26 for H-RARP). However, since LND did not significantly alter differences between platforms [33], it was excluded from the main analysis and addressed separately trough a subgroup analysis in the results. Since the main objective of this paper is to compare the two robotic platforms, this aspect was explicitly considered in the analysis.

The seventh step calculated the cost per each activity and the total cost per patient’s surgical procedure with the following cost equation:

1 $$C_{pt}\sum_i\beta_i(X_{j\backslash i}\cdot CCR_j)+y$$

where:

$C_{pt}$: total cost of the surgery for the patient *pt*;

*i*: activity considered (i.e. *Undocking*,*Undraping*, *Room configuration*, $\cdots$);

*j*: resource considered (i.e.: *Table Nurse*,*Senior surgeon*, $\cdots$);

$\beta_i$: time spent in the activity *i*;

$X_{j\backslash i}$: quantity of the resource *j* in the activity *i*;

*CCR*_*j*_: Cost Capacity Rate of the resource *j*;

*y*: other direct costs directly attributed to the procedure.

Direct costs appear as y in the time equation because they are allocated directly to the cost object, in our case the robotic kit and associated consumables. These direct costs are allocated to the entire operation without using time. Indirect costs (operating room, platform fee and maintenance) and a typology of direct costs (personnel) are allocated through time (β) to each mapped activity. Indirect costs are allocated to each activity by multiplying the cost-capacity ratio for each resource (CCR_j_) by the number of resources used in the activity (X_j|i_) (e.g., 2 nurses for the room configuration activity) and the time estimated trough chrono analysis (β) for the activity (i) (∑β_i_x_j|i_CCR_i_ ). The total cost for each single activity *i* is given by the sum of each resource (*j*) involved in that activity (for example for the prep and position activity we have *β [2.5 Nurses x CCR*_*Nurse*_*+ 1 Senior surgeon x CCR*_*senior surgeon*_*+ 1 Senior anesthesiologist x CCR*_*Senior anesthesiologist*_*+ 2 medical trainee x CCR*_*medical trainee*_*])*. The total cost is then computed summing the cost for each activity plus the direct cost (y) as in the formula (1).

Finally, the eighth step consisted of performing analytics, detailed in subsequent sections of the paper. Further analysis conducted for the learning curve associated with the Hugo platform. As documented by Antonelli et al. [33], Hugo requires an adjustment period and proficiency achieved after 17 cases for the console activity and 22 cases for prep and positioning. To incorporate this, cost differences between the first 22 and subsequent 28 H-RARP cases were evaluated, allowing for a comparison of costs before and after proficiency was achieved.

### Sensitivity analyses

A series of sensitivity analyses was conducted to evaluate the effect of parameter variability on total cost. First, one-way deterministic sensitivity analyses were performed by increasing and decreasing base case values by 30%. The results were visualized using a tornado diagram, which highlights the most sensitive parameters.

Next, threshold and two-way sensitivity analyses were conducted for the most influential parameters identified in the one-way analysis. These analyses aimed to determine the conditions under which the costs of the two platforms would converge and to explore how simultaneously changes in multiple parameters would affect the total operation cost.

## Results

Table 1 illustrates the total costs per operation and for each activity. The DV-RARP cost is statistically more expensive than H-RARP (DV-RARP = €4,979.21; H-RARP= €3,511.73; 42% difference; *p*-value < 0.001). However, when examining case-time cost, DV-RARP was less expensive than H-RARP (da Vinci = €1,481.18; Hugo = €1,926.00; 23% difference; *p*-value < 0.001), though this is offset by higher material costs (da Vinci = €3,498.03; Hugo = €1,586.00). The most costly activity, “surgery,” costs €1,043.00 for da Vinci and €1,370.00 for Hugo (24% difference; *p*-value < 0.001). Regarding the console activity, the cheaper platform is da Vinci with €927.40 and €1,259.00 for Hugo (26% difference; *p*-value < 0.001). “Prep and positioning” cost is €143.40 for da Vinci and €210.70 for Hugo (32% difference; *p*-value < 0.001). Lastly, the “room setup” cost amounts to €70.02 and €107.10 (34.65% difference; *p*-value < 0.001). No significant difference was found for the “anesthesia” activity because its cost for DV-RARP was €224.37 and €237.14 for H-RARP (6% difference; *p*-value = 0.5008).

**Table 1:** Prostatectomy procedure cost detailing (€) comparison between platforms

Activity and consumables	DA VINCI (*n* = 50)				HUGO (*n* = 50)				*p*.value*
	mean	sd	min	max	mean	sd	min	max	
***Room setup***	70.02	20.17	29.33	122.50	107.10	33.45	41.73	208.60	<0.001
***Anesthesia***	224.40	82.95	82.96	513.10	237.70	112.90	18.68	476.70	0.5008
***Prep and positioning***	143.40	65.61	74.86	538.60	210.70	60.33	104.00	360.30	<0.001
***Surgery (total)***	1,043.00	253.00	570.70	1,663.00	1,370.00	298.50	744.70	2,223.00	<0.001
*** Surgery (only console)***	927.40	230.50	513.50	1,606.00	1,259.00	287.40	658.20	2,102.00	<0.001
**Case time**	1,481.18	272.64	978.86	2,168.05	1,926.00	345.70	1,427.00	3,003.00	<0.001
**Kit Cost**	3,498.03	0	3,498.03	3,498.03	1,586.00	0	1,586.00	1,586.00	<0.001
**Total cost per operation**	4,979.21	272.64	4,476.89	5,666.08	3,511.73	345.71	3,012.93	4,588.52	<0.001

*P-value from t-test. The results do not vary when using Wilcoxon-Mann-Whitney

Analysis of the individual micro-activities on the map identified only a few activities without significant cost differences: “anesthesia,” “room configuration,” “patient positioning,” “trocar positioning,” “opening of the umbilical-prevesical fascia,” and “extraction of the operative specimen.” For all other micro-activities, DV-RARP was significantly less costly (*p*-value < 0.05). (Detailed results are provided in Table 2 in the Appendix)

**Fig. 2:**
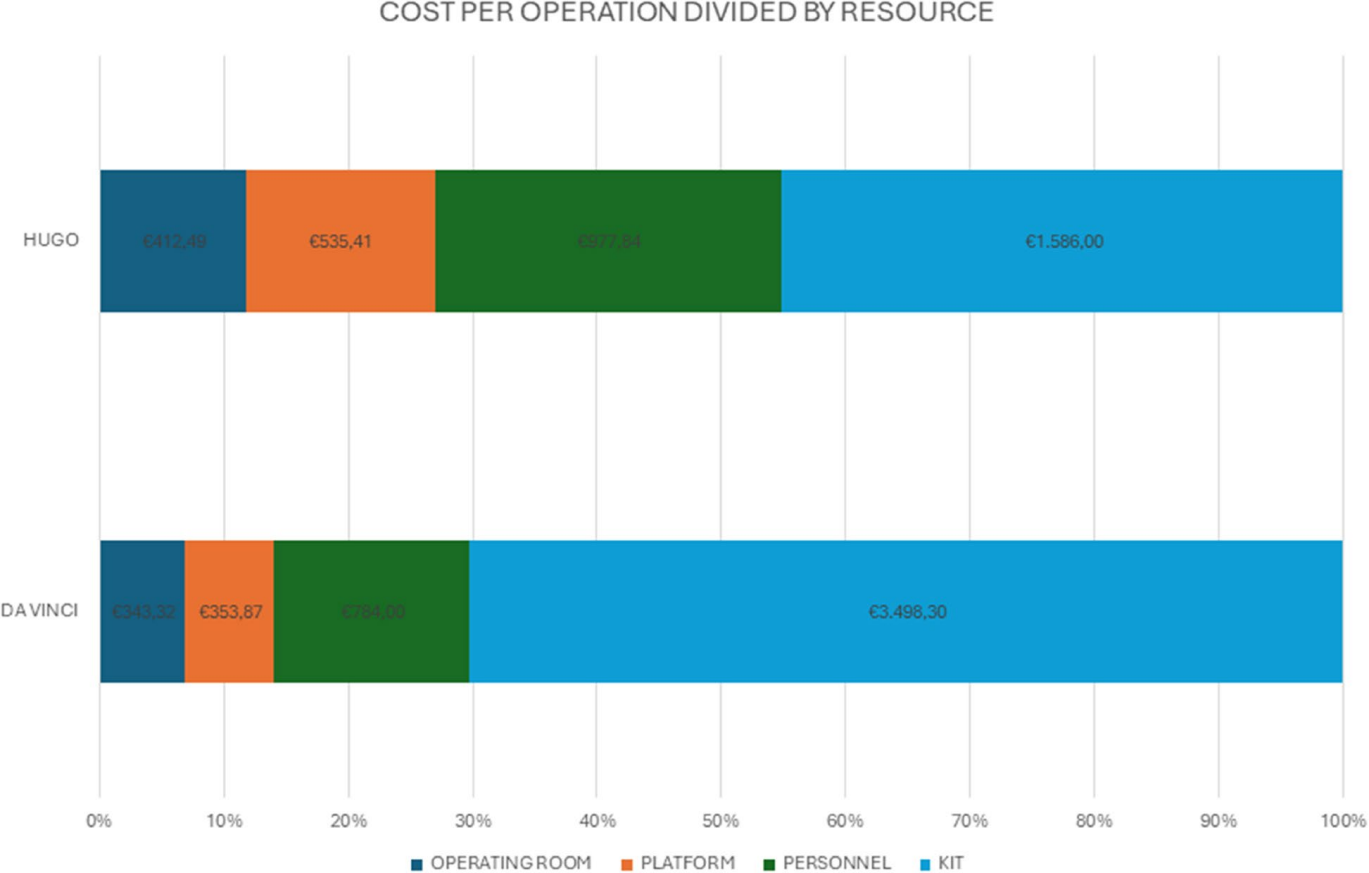
Composition of costs by resources

Figure 2 illustrates the cost breakdown by resource of both platforms. The surgical kit constituted the largest cost driver, representing 70.3% of total costs for DV-RARP and 45.2% for H-RARP. Personnel costs were €784.00 for DV-RARP (15.7% of total costs) and €977.80 for H-RARP (27.8%; *p*-value < 0.001). Platform costs were €353.90 (7.1%) for da Vinci and €535.40 for Hugo (15.2%; *p*-value < 0.001). Operating room costs were €343.30 (6.9%) for DV-RARP and €412.50 (11.7%; *p*-value < 0.001). Both the *t*-test and Wilcoxon test confirmed these findings, with the exception of non-significant differences in the “anesthesia” (see Table 3 in the Appendix).

Table 4 in Appendix show a sub-analysis for patients who underwent LND. Total time and costs were higher for these patients across both platforms. However, comparisons between robotic platforms revealed no significant differences for the LND (*p* = 0.643). This analysis reaffirms that DV-RARP remains less costly than H-RARP for the remaining activities.

As previously outlined in the methodology section, the analysis shifts to H-RARP to access cost differences between the first 22 cases (pre-proficiency) and the subsequent 28 cases. Table 2 indicates a reduction in costs for H-RARP by €358.45 (*p* < 0.001) between the last initial and later cases. Of this, €292.79 was attributed to the surgery activity (*p* < 0.001) and €44.95 to prep and positioning (*p* = 0.0075). Costs for the anesthesia and room setup activities demonstrated no significant differences. Despite the cost reduction, DV-RARP remained less expensive than H-RARP in case-time costs, and individual activities (see Table 2).

**Table 2:** Prostatectomy procedure cost detailing (€) for the comparison between first 22 cases of Hugo, last 28 of Hugo and all Da Vinci

Activity								
	HUGO (*n* = 22)		HUGO (*n* = 28)			DA VINCI (*n* = 50)		
	Mean cost	SD	Mean cost	SD	*p*.value	Mean cost	SD	*p*.value *
**Room setup**	111.70	39.111	103.57	28.49	0.3988	70.02	20.17	<0.001
**Anesthesia**	244.81	118.54	232.20	110.12	0.6992	224.36	82.95	0.7235
**Prep and positioning**	235.87	60.98	190.91	52.83	0.0075	143.36	65.61	0.0016
**Surgery (total)**	1,534.09	328.45	1,241.34	196.68	<0.001	1,043.44	253.02	<0.001
*** Surgery( only console)***	1,413.70	309.79	1,137.78	201.20	<0.001	927.45	230.49	<0.001
**Case time**	2,126.47	403.13	1,768.01	178.50	<0.001	1,481.18	272.64	<0.001
**Total cost per operation**	3,712.46	403.13	3,354.01	178.50	<0.001	4,979.21	272.64	<0.001

*P-value from t-test. The results do not vary when using Wilcoxon-Mann-Whitney

To evaluate the potential impact of postoperative length of stay on cost differences, *t*-tests were conducted. Table 3 illustrates no significant difference in postoperative length of stay between the platforms (DV-RARP: 4.52 days; H-RARP: 4.16 days; *p* = 0.065). Readmission rates were similarly low and one case reported for each platform. As a result, costs related to length of stay and readmission were not included in further analyses.

**Table 3:** Post operative length of stay per RARP

Variable	DA VINCI (*n* = 50)				HUGO (*n* = 50)				*p*.value*
	mean	sd	min	max	mean	sd	min	max	
***Post-operative length of stay***	4.52	1.12	3	9	4.16	0.76	3	6	0.065

*P-value from t-test. The results do not vary when using Wilcoxon-Mann-Whitney

### Sensitivity analyses

**Fig. 3:**
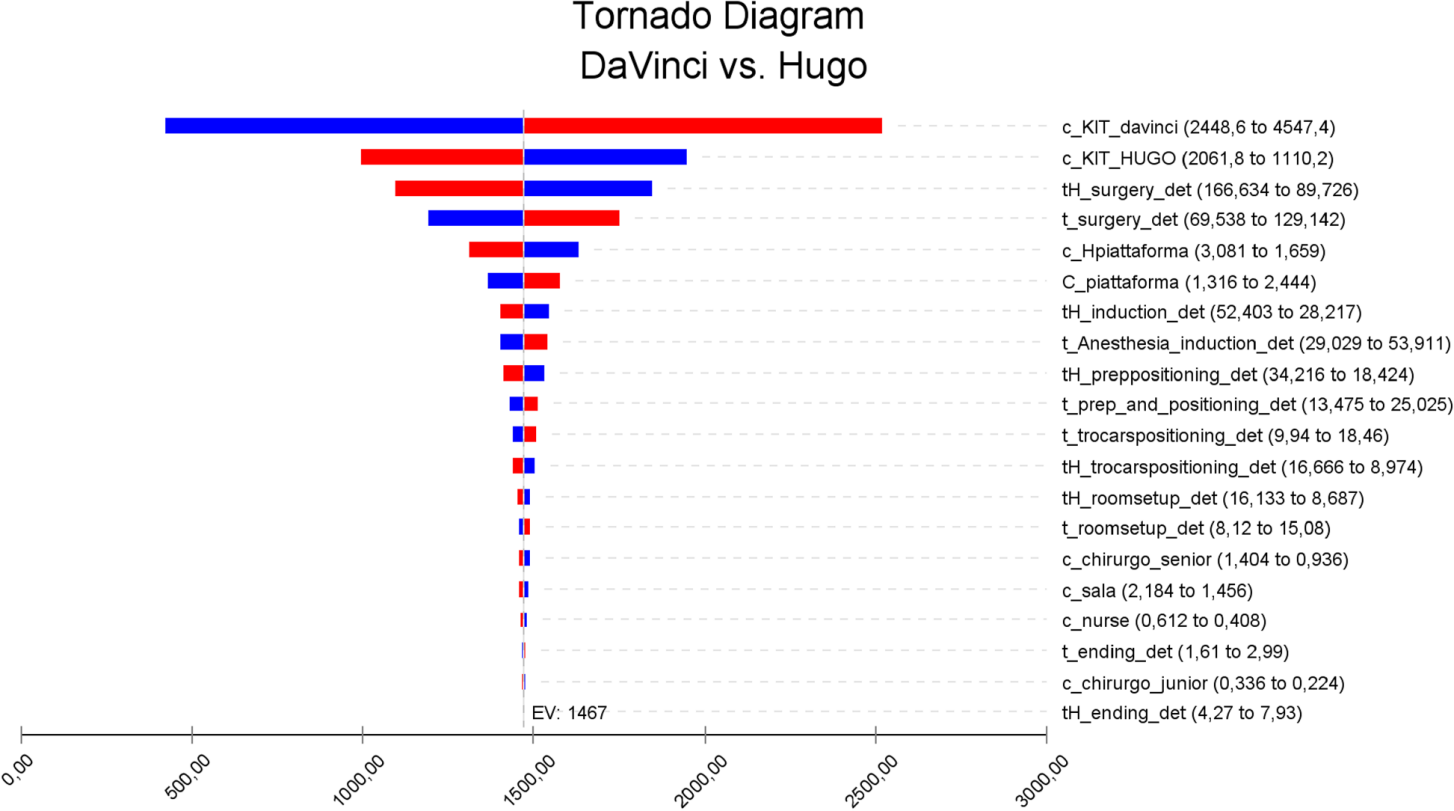
Deterministic sensitivity analysis tornado diagram. Legend: results of selective one-way sensitivity analysis in which several model input parameters were varied to determine their effect on the difference of costs between the two platforms. Blue bars represent the base-case input parameter values minus 30%. Red bars represent base case input parameter values plus 30%

The tornado diagram (Fig. 3) summarizes the one-way sensitivity analyses for the main cost parameters, arranged from most to least sensitive. The cost of the surgical kits emerged as the most critical parameter, particularly for da Vinci. A 30% change in da Vinci kit cost resulted in a €1,000 variation in the cost differential between platforms, compared with €600 for the Hugo kit.

Other sensitive parameters included surgery time for H-RARP, in which a 30% change caused a €400 cost difference, and the surgery time for DV-RARP, with a €300 impact. Platform rental fees also influenced costs; a 30% increase in the rental fee raised costs by €200. Similarly, a 30% change in the rental fee for da Vinci resulted in an approximate €100 change in the cost of DV-RARP. Across all one-way analysis, DV-RARP remained the more costly procedure.

**Fig. 4:**
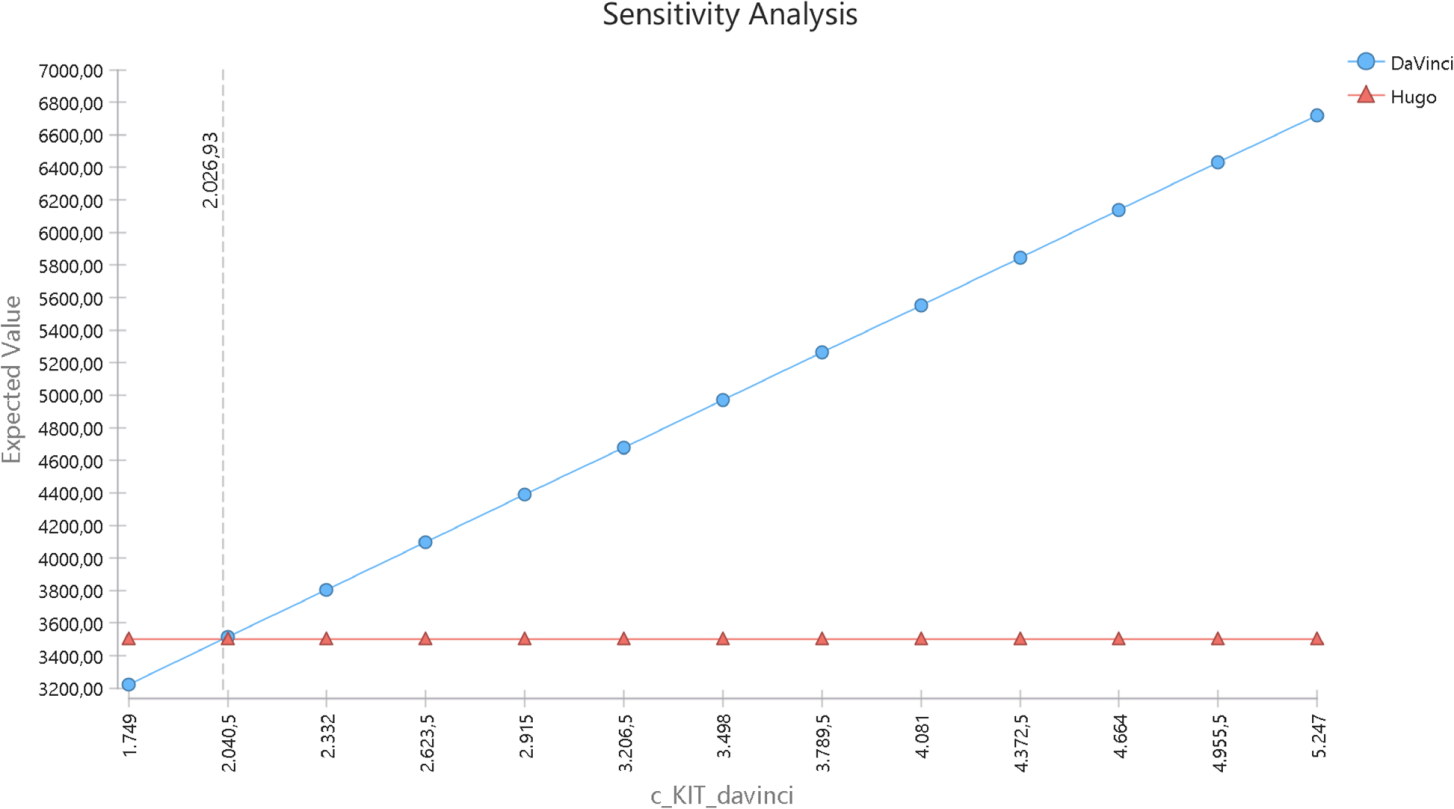
Threshold analysis for the cost of the Da Vinci’s kit

A threshold analysis revealed that cost parity between platforms could be achieved if the da Vinci kit cost was reduced to approximately €2,026.93 (Fig. 4).

**Fig. 5:**
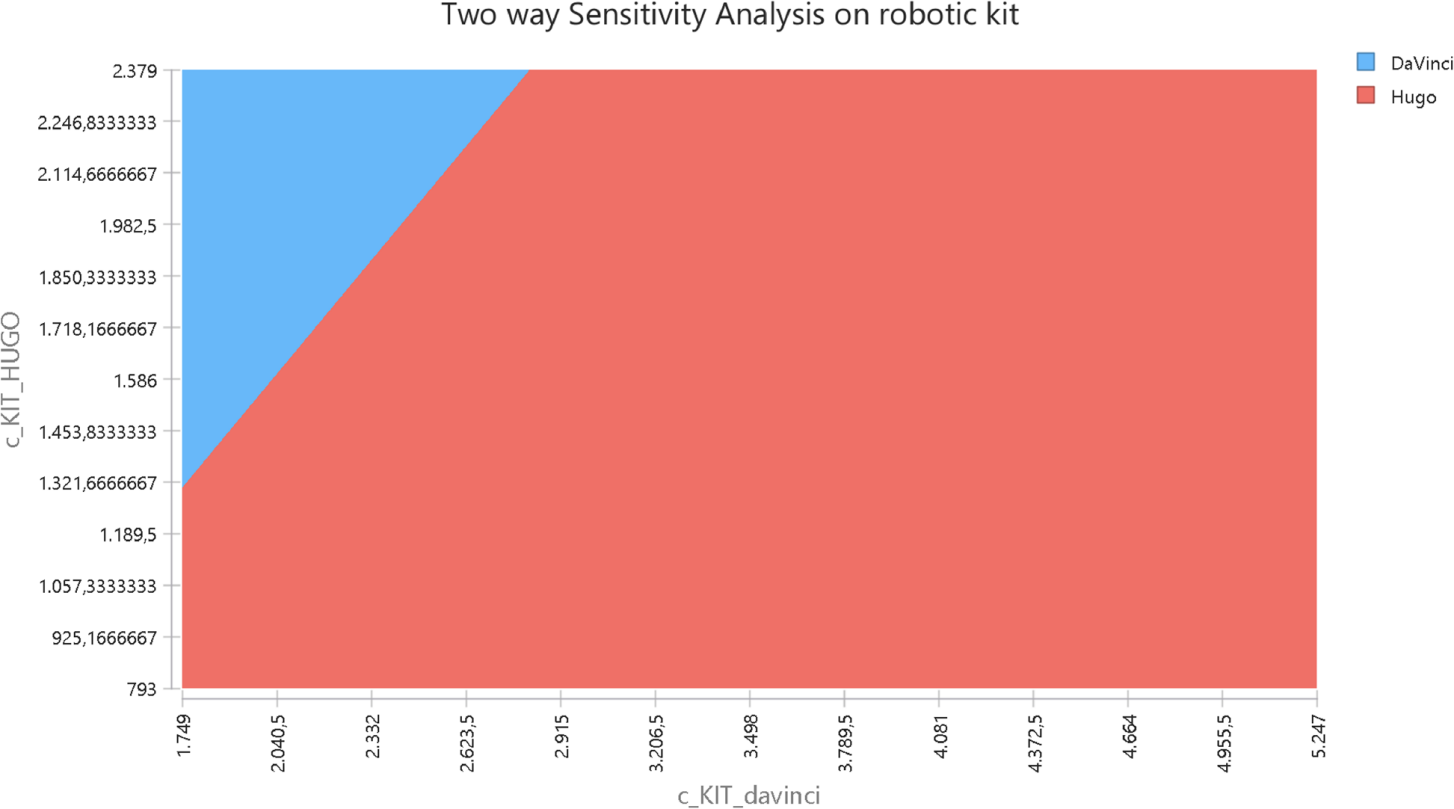
Two-way sensitivity analysis with both kits. Legend: the red area indicates where H-RARP is cheaper than DV-RARP. The blue area indicates where DV-RARP is cheaper than H-RARP

Figure 5 illustrates the cost equivalence frontier, depicting material price combinations that would result in equal operational cost. For example, if Hugo’s kit costs €1,321, the da Vinci’s kit must be priced at €1,749 for parity. Conversely, if the Hugo kit increases to €2,114, the da Vinci must cost €2,623.

As illustrated in the tornado diagram, the most cost-sensitive parameters were the surgical kits and rental fees. Subsequently, two-way sensitivity analyses were conducted for each platform to evaluate how changes in the cost of the kit and rental of the platform affect the cost of DV-RARP and H-RARP. These analyses are presented in matrix form (Tables 5 and 6 respectively in the Appendix) and reveal cost variations with rental fees ranging from − 30% to + 30% (left to right) and kit costs ranging from + 30% to − 30% (top to bottom).

A simultaneous 30% reduction in the kit and rental fee for DV-RARP results in a total cost of €3,823.80. Nevertheless, this figure remains higher than the baseline cost of H-RARP (€3,511.74). Conversely, a 30% increase to the kit cost and rental fee for H-RARP results in a total cost of €4,148.16, which is still lower than the baseline cost of DV-RARP (€4,979.49). These results confirm the tornado diagram findings, highlighting the critical sensitivity of kit costs.

Notably, a reduction in the kit cost alone by as little as 10% consistently reduces the total cost of RARP, even when rental fees are increased. The sole exception occurs with H-RARP when a 10% reduction in kit costs is combined with a 30% increase in rental fees, resulting in only a negligible cost difference of approximately €2.00.

## Discussion and conclusion

In the complex decision-making landscape of the top management of healthcare organizations, the introduction of disruptive technologies presents significant challenges. While robotic surgery has become a transformative and indispensable tool in healthcare evolution [41], its introduction requires managers to strike a balance between the professionals’ needs and the administrative imperatives of efficient resource management. The recent democratization of robotics in healthcare [17] has further intensified the need to find this equilibrium, leveraging appropriate tools to assess the degree of suitability of each platform.

In this context, this study contributes to the literature pointing out how the implementation of TDABC (and related sensitivity analyses), beyond its role in monitoring the cost of activities and optimizing the activities’ timing [42], serves as a strategic tool to support the decision-making process of hospitals’ organizational apex along a VBHC perspective, providing a suitable interpretation of value creation [29]. While previous literature recognized that TDABC is the most appropriate tool for implementing VBHC [28, 29], its robotic surgery-related implementations have been confined primarily to comparative cost analyses of robotic assisted surgery versus traditional laparoscopic or open surgical approaches (e.g., [43]). Nonetheless, the case of the robotic surgery trial conducted at the AOUIVR serves as a compelling example of how TDABC can effectively bridge the inherent tension between clinical needs and administrative constraints within the professional bureaucracy structure of a healthcare organization [15]. Indeed, by actively involving clinicians in this process, TDABC leverages their specialized knowledge to inform strategic decision-making, also providing unique insights into the value generated by each robotic platform at any stage of surgical procedures. This facilitates a more informed decision on how one robotic platform produces more value than others.

In line with VBHC, it is essential to investigate the dollar spent per clinical outcome, as defined by Porter [24]. This study applied TDABC to analyze the cost component of Porter’s value formula [24] when evaluating RARP conducted using two different robotic platforms: Da Vinci and Hugo. The findings demonstrate that while clinical outcomes might be comparable, the costs associated with each platform can differ because of differences in terms of time spent on various surgical steps and the cost of the robotic kits. Specifically, our findings reveal that although the intraoperative factor [33] and postoperative outcomes—such as readmission and length of stay [35]—are comparable between the two platforms, H-RARP tends to be less expensive than DV-RARP.

A closer examination of the micro-costing activity revealed insights into where these differences occur and identified opportunities for improvements. For instance, when we excluded the costs of the robotic kits and focused solely on the time-based costs, DV-RARP emerged as the highest-value option. The TDABC comparative analysis highlights that, across the different activities of the surgical process [30], the total cost of case-time for DV-RARP was significantly lower than that for H-RARP. Specifically, excluding the anesthesia, DV-RARP consistently demonstrated lower costs across all other surgical process stages than H-RARP. This finding can be ascribed to the team’s familiarity with the da Vinci system, which has been the gold standard in robotic surgery for nearly two decades [18, 19]. Notably, this result held even when considering the team’s learning curve for the Hugo platform, which Antonelli et al. [33] found to reach a first plateau (i.e., proficiency) after 22 examples.

Regarding costs, excluding the cost of the surgical kit, personnel expenses constitute the most significant expenditure. This finding aligns with previous research on the implementation of robotics in surgery (e.g., [44]), highlighting the substantial personnel time and costs associated with these procedures. The presence of multiple professionals during operations, particularly the requirement for two senior surgeons during the time-intensive console step, contributes significantly to these elevated costs. The findings are further supported by sensitivity analyses that identified the duration of the surgery time—as the longer and most resource-consuming activity in terms of personnel—as the most sensitive parameter influencing costs, right after the surgical kit.

A particularly relevant observation within this analysis pertains to the cost differentials across various surgical activities. While room setup constituted the least costly part, it exhibited the most significant relative cost difference (34.2%) compared with others. This disparity can be attributed to the team’s familiarity with the specific robotic platform because proficiency in setting up the equipment can have a significant impact. Conversely, the anesthesia activities exhibited no significant cost differences between the two platforms. This observation can be attributed to the fact that the robotic platforms were not actively used during anesthesia.

The insights from this article also contribute to the practitioner knowledge base and the applied methodology. For instance, sensitivity analysis alongside the TDABC allowed us to highlight the pivotal role of kit costs in shaping the overall operational expenses. The cost of the surgical kit emerged as a primary driver of the cost differential between DV-RARP and H-RARP. In detail, findings reveal that assuming all other factors remain constant, cost parity between the two platforms could be achieved with a reduction of approximately €2,000 in the DV-RARP kit cost.

Our results demonstrate a way to exploit the characteristics of the micro-costing method TDABC and related sensitivity analyses to support managerial decision-making regarding the purchase of new robotics platforms. In contrast to methods used in previous literature (e.g., [21]). TDABC enables the calculation of the cost of an operation per individual patient [29]. Furthermore, as already shown, it allows the assessment of the value generated for each activity [30]. Additionally, through the sensitivity analyses conducted, the impact of each component on the total cost was assessed, identifying those parameters on which managers may be able to negotiate with vendors and improve the process efficiency.

### Limitations and future research

Despite the contributions mentioned above, it is important to recognize certain caveats and directions for future studies. First, our study is based on data at the patient level for resource use, whereas data for the robotic platforms were specific to the tender developed by the hospital to carry out the clinical trial presented here. In addition, we had data on significant amounts of material for the tender, and not on the individual material. Even though we partially circumvented this constraint by employing sensitivity analysis, future research on the same topic could provide different points of view considering data from routine tenders or from more than one tender. In any case, it is important to survey sensitivity analysis because the prices of the platforms and materials are ever-changing according to several factors. Future research should consider this fact and provide sensitivity analysis to generalize the results to other settings.

The second aspect that future research could investigate pertains to the effect of proficiency. Following the proficiency threshold identified in previous literature in H-RARP (i.e., 22 cases [33]) it can be contended that the previous DV-RARPs performed by surgeons (> 500 cases) [33] and the team’s overall familiarity played a relevant role in the procedures. Future research could use the methodology applied in this paper in a comparable organizational context, where the team has similar experiences with both platforms.

In addition, a full assessment of the two robotic platforms, which was beyond this paper’s scope, should consider the multidimensionality of health technology assessment and examine more than just one surgical procedure. While robotic platforms are particularly relevant in the field of urology (and, more specifically, radical prostatectomy [5, 6]), following the recommendation of Erskine et al. [45] to properly assess the value that one platform can deliver, future research endeavors could focus on the robotic application in different procedures.

Finally, among other dimensions such as organizational, legal, social, or ergonomic aspects for surgeons (e.g., [12]), there has been a growing concern over the environmental sustainability of hospitals (e.g., [46]), and operating rooms (see [47]). The growing demand for comprehensive evaluations has created a greater need to conduct cost, clinical evaluations, and environmental assessments. Previous research has demonstrated that robotic surgery has lower carbon dioxide emissions than laparoscopic surgery [48]. Future research should explore the environmental impact associated with each platform and determine whether there are any substantial differences.

In conclusion, this study compared two robotic platforms used in RARP through the TDABC method. Although clinical outcomes were reported to be similar, the Hugo platform is less expensive overall, whereas the da Vinci platform offers greater value when excluding kit costs. The methodology used provides insights for hospital administrators to optimize decision-making and vendor negotiations. Additionally, it serves as a useful framework for future research on the comparative cost-effectiveness of robotic platforms in healthcare.

## Supplementary Information

Supplementary Material 1.

## Data Availability

The data that support the findings of this study are available from Azienda Ospedaliera Universitaria Integrata Verona, but restrictions apply to the availability of these data, which were used under license for the current study, and so are not publicly available. The data are, however, available from the authors upon reasonable request and with the permission of Azienda Ospedaliera Universitaria Integrata Verona.

## Abbreviations

RARP: Robot-assisted radical prostatectomy
DV-RARP: Robot-assisted radical prostatectomy performed with da Vinci platform
H-RARP: Robot-assisted radical prostatectomy performed with Hugo platform
VBHC: Value-based healthcare
TDABC: Time-driven activity-based costing
AOUIVR: Azienda Universitaria Integrata di Verona
CDVC: Care delivery value chain
CCR: Capacity cost rate
LND: Lymph node dissection
RAS: robotic-assisted surgery
