# Supplementary material for: Supporting managerial decisions: a comparison of new robotic platforms through time-driven activity-based costing within a value-based healthcare framework
Source: BMC Health Serv Res. 2025 Mar 29;25:470. doi: 10.1186/s12913-025-12598-9 (PMC11954269; doi:10.1186/s12913-025-12598-9)
Supplement: Supplementary file 1 — Supplementary Material 1. [file 12913_2025_12598_MOESM1_ESM.docx]

**APPENDIX**

| Table 1. List of resources used during RARP procedure and relative CCR . “X” indicates the activity where the resource is present. | | | | |  |  |  |
| --- | --- | --- | --- | --- | --- | --- | --- |
| **Unit** | **Cost allocated to unit per year** | **CCRs (€)** | **Room setup** | **Anesthesia** | **Prep and positioning** | **Surgery (total)** | **Surgery (console)** |
| **Theather related** | Platform DV | 1.88 | x | x | x | x | x |
|  | Da Vinci's kit (Maryland pliers; Trocar Air Seal;Retrieval System…) | 3,498 |  |  |  |  |  |
|  | Platform Hugo | 2.36 | x | x | x | x | x |
|  | Hugo's kit  (monopolar curved forceps;ladder jack...) | 1,073 |  |  |  |  |  |
|  | Operating room  (Depreciation, Energy, Mainentance, Non-healthcare services, healthcare services,rental fees) | 1.82 | x | x | x | x | x |
| **Human related** | Senior surgeon | 1.17 | 0 | 0 | 1 | 1 | 2 |
|  | Senior anesthesiologist | 1.17 | 0 | 1 | 1 | 1 | 1 |
|  | Surgeon in training | 0.28 | 0 | 0 | 1 | 1 | 1 |
|  | Anesthesiologist in training | 0.28 | 0 | 1 | 1 | 1 | 1 |
|  | Table nurse | 0.51 | 1 | 1 | 1 | 1 | 1 |
|  | Instrument nurse | 0.51 | 1 | 1 | 1 | 1 | 1 |
|  | Anesthesiologist nurse | 0.51 | 0 | 0.5 | 0.5 | 0.5 | 0.5 |

| Table 2. Mean cost (€) for micro-activity | | |  |  |  |  |  |  |  |  |  |  |  |
| --- | --- | --- | --- | --- | --- | --- | --- | --- | --- | --- | --- | --- | --- |
| ACTIVITY |  | |  |  |  |  |  |  |  |  |  |  |  |
|  |  | | DA VINCI (n =50) | | | |  | HUGO (n =50) | | | |  |  |
|  |  | | mean | sd | min | max |  | mean | sd | min | max |  | p.value* |
|  |  | |  |  |  |  |  |  |  |  |  |  |  |
| Room configuration | |  | 45.44 | 19.61 | 7.88 | 103.30 |  | 41.44 | 20.72 | 5.21 | 93.92 |  | 0.3234 |
| Draping of platform | |  | 9.59 | 7.67 | 3.22 | 36.27 |  | 23.32 | 9.05 | 5.21 | 40.00 |  | 0 |
| Anesthesia induction | |  | 224.43 | 82.95 | 82.96 | 513.10 |  | 237.71 | 112.90 | 18.68 | 476.70 |  | 0.5008 |
| Patient position | |  | 18.76 | 8.55 | 6.47 | 51.70 |  | 21.47 | 14.38 | 3.47 | 86.87 |  | 0.2542 |
| Draping of the patient and cables connection | |  | 20.74 | 6.94 | 6.46 | 38.77 |  | 28.01 | 10.68 | 6.95 | 62.55 |  | <0.0001 |
| Trocars placement | |  | 116.00 | 102.81 | 16.34 | 465.60 |  | 111.01 | 65.66 | 43.28 | 424.10 |  | 0.7715 |
| Docking | |  | 46.40 | 14.62 | 16.34 | 106.20 |  | 100.20 | 44.14 | 34.62 | 242.40 |  | 0 |
| Opening of the umbilical-prevesical fascia | |  | 110.00 | 66.99 | 28.01 | 354.82 |  | 96.47 | 59.26 | 19.65 | 324.20 |  | 0.288 |
| Preparation of the Retzius space | |  | 65.54 | 54.49 | 18.67 | 308.10 |  | 87.63 | 36.04 | 19.65 | 186.60 |  | 0.0187 |
| Dissection of the bladder neck | |  | 101.20 | 43.89 | 28.01 | 252.10 |  | 164.20 | 80.91 | 49.12 | 383.10 |  | 0 |
| Dissection of the seminal vesicles | |  | 112.00 | 57.02 | 18.67 | 242.70 |  | 193.90 | 116.30 | 39.29 | 687.60 |  | 0 |
| Dissection of the posterior plane | |  | 50.60 | 35.29 | 9.34 | 149.40 |  | 68.57 | 46.44 | 19.65 | 245.60 |  | 0.0318 |
| Management of prostatic pedicles and eventual nerve sparing | |  | 98.22 | 56.02 | 18.67 | 252.10 |  | 128.30 | 67.90 | 19.65 | 294.70 |  | 0.0175 |
| Dissection of the prostate apex and urethra | |  | 98.59 | 35.04 | 46.68 | 196.10 |  | 141.50 | 94.91 | 29.47 | 540.30 |  | 0.0035 |
| Posterior reconstruction and urethrovesical anastomosis | |  | 291.30 | 93.28 | 121.40 | 569.50 |  | 378.60 | 164.30 | 98.24 | 854.60 |  | 0.0015 |
| Extraction of the operative specimen | |  | 44.27 | 62.06 | 8.62 | 441.10 |  | 40.68 | 26.13 | 8.65 | 129.80 |  | 0.707 |
| Undocking | |  | 13.18 | 8.14 | 6.46 | 45.24 |  | 20.29 | 12.86 | 6.95 | 83.40 |  | 0.0013 |
| Undraping | |  | 14.99 | 6.19 | 6.46 | 32.310 |  | 42.39 | 26.16 | 6.95 | 118.10 |  | 0 |
|  |  | |  |  |  |  |  |  |  |  |  |  |  |

*P-value from t-test. The results do not vary when using Wilcoxon-Mann-Whitney

| Table 3. Costs (€) for activity and resources | | |  |  |  |  |  |  |  |  |  |  |  |  |  |
| --- | --- | --- | --- | --- | --- | --- | --- | --- | --- | --- | --- | --- | --- | --- | --- |
| **PHASE** | PLATFORM | | | | | OPERATING ROOM | | | | | PERSONNEL | | | | |
|  | mean | sd | min | max | p-value* | mean | sd | min | max | p-value* | mean | sd | min | max | p-value* |
| **ROOMSETUP** |  |  |  |  |  |  |  |  |  |  |  |  |  |  |  |
| Da Vinci (n= 50) | 26.23 | 7.85 | 10.97 | 47.32 | <0.0001 | 25.45 | 7.61 | 10.64 | 45.91 | <0.0001 | 18.34 | 4.92 | 7.72 | 30.34 | <0.0001 |
| Hugo (n=50) | 43.82 | 12.98 | 18.15 | 81.29 |  | 33.76 | 10.00 | 13.98 | 62.63 |  | 29.56 | 11.18 | 9.60 | 64.67 |  |
| **ANESTHESIA** |  |  |  |  |  |  |  |  |  |  |  |  |  |  |  |
| Da Vinci (n= 50) | 77.96 | 28.83 | 28.83 | 178.30 | 0.0235 | 75.64 | 27.97 | 27.97 | 173.00 | 0.7393 | 70.75 | 26.16 | 26.16 | 161.80 | 0.7393 |
| Hugo (n=50) | 95.44 | 45.32 | 7.50 | 191.40 |  | 73.53 | 34.91 | 5.78 | 147.40 |  | 68.78 | 32.66 | 5.40 | 137.90 |  |
| **PREPANDPOSITIONING** |  |  |  |  |  |  |  |  |  |  |  |  |  |  |  |
| Da Vinci (n= 50) | 36.20 | 15.29 | 18.80 | 126.90 | <0.0001 | 35.12 | 14.84 | 18.24 | 123.10 | <0.0001 | 72.04 | 35.58 | 37.82 | 288.50 | <0.0001 |
| Hugo (n=50) | 62.31 | 17.33 | 30.78 | 103.00 |  | 48.01 | 13.35 | 23.71 | 79.35 |  | 100.40 | 29.84 | 49.50 | 178.00 |  |
| **SURGERY (total)** |  |  |  |  |  |  |  |  |  |  |  |  |  |  |  |
| Da Vinci (n= 50) | 213.50 | 52.25 | 116.60 | 336.50 | <0.0001 | 207.10 | 50.69 | 113.10 | 326.50 | <0.0001 | 622.90 | 150.30 | 341.00 | 999.90 | <0.0001 |
| Hugo (n=50) | 333.80 | 72.56 | 182.30 | 539.80 |  | 257.20 | 55.90 | 140.50 | 415.90 |  | 779.10 | 170.10 | 422.00 | 1,268.00 |  |
| ***Surgery (only console)*** |  |  |  |  |  |  |  |  |  |  |  |  |  |  |  |
| Da Vinci (n= 50) | 186.80 | 46.42 | 103.40 | 323.40 | <0.0001 | 181.20 | 45.03 | 100.30 | 313.70 | <0.0001 | 559.50 | 139.00 | 309.80 | 968.70 | <0.0001 |
| Hugo (n=50) | 303.50 | 69.26 | 158.60 | 506.70 |  | 233.80 | 53.36 | 122.20 | 390.30 |  | 721.90 | 164.70 | 377.30 | 1,205.00 |  |
| **CASE TIME** |  |  |  |  |  |  |  |  |  |  |  |  |  |  |  |
| Da Vinci (n= 50) | 353.90 | 61.57 | 242.50 | 498.20 | <0.0001 | 343.30 | 59.74 | 235.30 | 483.40 | <0.0001 | 784.00 | 155.40 | 501.00 | 1,186.00 | <0.0001 |
| Hugo (n=50) | 535.40 | 92.26 | 395.40 | 818.40 |  | 412.50 | 71.08 | 304.60 | 630.50 |  | 977.80 | 186.90 | 653.70 | 1,554.00 |  |
|  |  |  |  |  |  |  |  |  |  |  |  |  |  |  |  |

*P-value from t-test. The results do not vary when using Wilcoxon-Mann-Whitney

| Table 4. Mean cost (€) of phases for patient undergoing LND | | | | | | | | | | | | |
| --- | --- | --- | --- | --- | --- | --- | --- | --- | --- | --- | --- | --- |
|  |  |  |  |  |  |  |  |  |  |  |  |  |
| ACTIVITY |  |  |  |  |  |  |  |  |  |  |  |  |
|  |  | DA VINCI (n =23) | | | |  | HUGO (n =26) | | | |  |  |
|  |  | mean | sd | min | max |  | mean | sd | min | max |  | p.value* |
| **ROOMSETUP** | | 69.2 | 19.7 | 29.3 | 121.1 |  | 107.2 | 39.0 | 41.7 | 208.6 |  | <0.0001 |
| **ANESTHESIA** | | 240.8 | 101.4 | 82.9 | 513.1 |  | 223.1 | 106.3 | 21.6 | 426.6 |  | 0.5542 |
| **PREPANDPOSITIONING** | | 149.9 | 88.5 | 92.7 | 538.6 |  | 214.2 | 62.4 | 128.3 | 360.3 |  | 0.0047 |
| **SURGERY (total)** | | 1,457 | 267.3 | 1,029 | 2,130 |  | 1,763 | 392.1 | 987.1 | 2,607 |  | 0.0029 |
| ***Surgery( only console)*** | | 1,339 | 265.5 | 939.4 | 2,064 |  | 1,669 | 397.1 | 900.5 | 2,547 |  | 0.0015 |
| ***LND*** |  | 460.3 | 154.2 | 187.9 | 776.0 |  | 484.4 | 201.8 | 233.7 | 1,004 |  | 0.6438 |
| **CASE TIME** | | 1,917 | 306.9 | 1,356 | 2,544 |  | 2,307 | 392.5 | 1,676 | 3,320 |  | 0.0004 |
| **TOTAL COST PER OPERATION** | | 5,415 | 306.9 | 4,854 | 6,042 |  | 3,893 | 392.5 | 3,262 | 4,906 |  | <0.0001 |

*P-value from t-test. The results do not vary when using Wilcoxon-Mann-Whitney

Table 5. Variation of DV-RARP cost based on rental fee and kit changes. The red square indicates the highest cost for the procedure. The green square the lowest.

|  |  | **VARIATION OF DA VICNCI'S RENTAL FEE** | | | | | | |
| --- | --- | --- | --- | --- | --- | --- | --- | --- |
|  |  | **-30%** | **-20%** | **-10%** | **BASELINE** | **10%** | **20%** | **30%** |
| **VARIATION OF DA VINCI'S KIT COST** | **30%** | 5,922.82 € | 5,958.20 € | 5,993.59 € | 6,028.98 € | 6,064.37 € | 6,099.75 € | 6,135.14 € |
|  | **20%** | 5,572.99 € | 5,608.37 € | 5,643.76 € | 5,679.15 € | 5,714.54 € | 5,749.92 € | 5,785.31 € |
|  | **10%** | 5,223.16 € | 5,258.54 € | 5,293.93 € | 5,329.32 € | 5,364.71 € | 5,400.09 € | 5,435.48 € |
|  | **BASELINE** | 4,873.33 € | 4,908.71 € | 4,944.10 € | **4,979.49 €** | 5,014.88 € | 5,050.26 € | 5,085.65 € |
|  | **-10%** | 4,523.50 € | 4,558.88 € | 4,594.27 € | 4,629.66 € | 4,665.05 € | 4,700.43 € | 4,735.82 € |
|  | **-20%** | 4,173.67 € | 4,209.05 € | 4,244.44 € | 4,279.83 € | 4,315.22 € | 4,350.60 € | 4,385.99 € |
|  | **-30%** | 3,823.84 € | 3,859.22 € | 3,894.61 € | 3,930.00 € | 3,965.39 € | 4,000.77 € | 4,036.16 € |

Table 6. Variation of H-RARP cost based on rental fee and kit changes. The red square indicates the highest cost for the procedure. The green square the lowest.

|  |  | **VARIATION OF HUGO'S RENTAL FEE** | | | | | | |
| --- | --- | --- | --- | --- | --- | --- | --- | --- |
|  |  | **-30%** | **-20%** | **-10%** | **BASELINE** | **10%** | **20%** | **30%** |
| **VARIATION OF HUGO'S KIT COST** | **30%** | 3,826.92 € | 3,880.46 € | 3,934.00 € | 3,987.54 € | 4,041.08 € | 4,094.62 € | 4,148.16 € |
|  | **20%** | 3,668.32 € | 3,721.86 € | 3,775.40 € | 3,828.94 € | 3,882.48 € | 3,936.02 € | 3,989.56 € |
|  | **10%** | 3,509.72 € | 3,563.26 € | 3,616.80 € | 3,670.34 € | 3,723.88 € | 3,777.42 € | 3,830.96 € |
|  | **BASELINE** | 3,351.12 € | 3,404.66 € | 3,458.20 € | **3,511.74 €** | 3,565.28 € | 3,618.82 € | 3,672.36 € |
|  | **-10%** | 3,192.52 € | 3,246.06 € | 3,299.60 € | 3,353.14 € | 3,406.68 € | 3,460.22 € | 3,513.76 € |
|  | **-20%** | 3,033.92 € | 3,087.46 € | 3,141.00 € | 3,194.54 € | 3,248.08 € | 3,301.62 € | 3,355.16 € |
|  | **-30%** | 2,875.32 € | 2,928.86 € | 2,982.40 € | 3,035.94 € | 3,089.48 € | 3,143.02 € | 3,196.56 € |
